# Near-wall hemodynamic changes in subclavian artery perfusion induced by retrograde inner branched thoracic endograft implantation

**DOI:** 10.1016/j.jvssci.2023.100116

**Published:** 2023-06-16

**Authors:** William J. Yoon, Kevin Mani, Sukgu M. Han, Cheong J. Lee, Jae S. Cho, Anders Wanhainen

**Affiliations:** a Department of Surgical Sciences, Vascular Surgery, Uppsala University, Uppsala, Sweden; b Division of Vascular Surgery and Endovascular Therapy, Department of Surgery, Case Western Reserve University School of Medicine, Cleveland, OH; c Comprehensive Aortic Center, Keck Medical Center of University of Southern California, Los Angeles, CA; d Division of Vascular Surgery, Department of Surgery, NorthShore University Health System, Evanston, IL; e Department of Surgical and perioperative Sciences, Surgery, Umeå University, Umeå, Sweden

**Keywords:** Branched endografts, Computational fluid dynamics, Left subclavian artery revascularization, Wall shear stress

## Abstract

**Objective:**

Left subclavian artery (LSA)-branched endografts with retrograde inner branch configuration (thoracic branch endoprosthesis [TBE]) offer a complete endovascular solution when LSA preservation is required during zone 2 thoracic endovascular aortic repair. However, the hemodynamic consequences of the TBE have not been well-investigated. We compared near-wall hemodynamic parameters before and after the TBE implantation using computational fluid dynamic simulations.

**Methods:**

Eleven patients who had undergone TBE implantation were included. Three-dimensional aortic arch geometries were constructed from the pre- and post-TBE implantation computed tomography images. The resulting 22 three-dimensional aortic arch geometries were then discretized into finite element meshes for computational fluid dynamic simulations. Inflow boundary conditions were prescribed using normal physiological pulsatile circulation. Outlet boundary conditions consisted of Windkessel models with previously published values. Blood flow, modeled as Newtonian fluid, simulations were performed with rigid wall assumptions using SimVascular's incompressible Navier-Stokes solver. We compared well-established hemodynamic descriptors: pressure, flow rate, time-averaged wall shear stress (TAWSS), the oscillatory shear index (OSI), and percent area with an OSI of >0.2. Data were presented on the stented portion of the LSA.

**Results:**

TBE implantation was associated with a small decrease in peak LSA pressure (153 mm Hg; interquartile range [IQR], 151-154 mm Hg vs 159 mm Hg; IQR, 158-160 mm Hg; *P* = .005). No difference was observed in peak LSA flow rates before and after implantation: 40.4 cm^3^/ (IQR, 39.5-41.6 cm^3^/s) vs 41.3 cm^3^/s (IQR, 37.2-44.8 cm^3^/s; *P* = .59). There was a significant postimplantation increase in TAWSS (15.2 dynes/cm^2^ [IQR, 12.2-17.7 dynes/cm^2^] vs 6.2 dynes/cm^2^ [IQR, 5.7-10.3 dynes/cm^2^]; *P* = .003), leading to decreases in both the OSI (0.088 [IQR, 0.063 to –0.099] vs 0.1 [IQR, 0.096-0.16]; *P* = .03) and percentage of area with an OSI of >0.2 (10.4 [IQR, 5.8-15.8] vs 15.7 [IQR, 10.7-31.9]; *P* = .13). Neither LSA side branch angulation (median, 81°, IQR, 77°-109°) nor moderate compression (16%-58%) seemed to have an impact on the pressure, flow rate, TAWSS, or percentage of area with an OSI of >0.2 in the stented LSA.

**Conclusions:**

The implantation of TBE produces modest hemodynamic disturbances that are unlikely to result in clinically relevant changes.

## Clinical Relevance

The Gore thoracic branch endoprosthesis represents the only commercially available stent graft in the United States for the treatment of aortic arch pathologies involving the left subclavian artery. The implantation of endografts cause alterations in flow patterns that, in turn, can have a significant impact on long-term device durability and the prognosis of the patient. The results of this computational modelling study provide important data regarding the hemodynamic performance of the thoracic branch endoprosthesis relevant to the associated clinical outcomes and subsequent implications on device durability.

**Article Highlights**

• **Type of Research:** Computational flow simulation study
• **Key Findings:** Left subclavian artery (LSA)-branched endograft (thoracic branch endoprosthesis) implantation resulted in a small but statistically significant decrease in the LSA pressure and did not cause a significant change in the LSA flow rate. LSA side branch angulation (range, 58°-157°) had no impact on the LSA pressure or flow rate.
• **Take Home Message:** Near-wall hemodynamic changes produced by the LSA-branched endograft with retrograde inner branch orientation are unlikely to be clinically significant.

A major limitation of thoracic endovascular aortic repair (TEVAR) for aortic arch pathology has been the presence of supra-aortic branch vessels in intended sealing zones.^1^^,^^2^ Because of the need for an adequate proximal seal zone for durable outcomes, the left subclavian artery (LSA) may need to be covered in ≤40% of patients undergoing zone 2 TEVAR.^3^^,^^4^ However, disruption of the LSA collaterals can cause significant complications, including stroke, spinal cord ischemia, and left upper extremity ischemia.^5^^,^^6^ As such, the risk of these complications associated with LSA coverage has been major concern.

To maintain LSA perfusion, several techniques have been developed, including open surgical revascularization of the LSA with extra-anatomical bypass grafting^7^ and a complete endovascular solution using parallel stent grafting (chimneys, snorkels, and periscopes)^8^^,^^9^ or in situ fenestration of standard aortic endografts.^10^^,^^11^ All these techniques have drawbacks and limitations, and no one technique is universally accepted. Thus, there is a need for a branched endograft designed for aortic arch pathology. An LSA branched endograft with retrograde portal or inner branch configuration, the TBE, has recently become commercially available with US Food and Drug Administration approval.^12^^,^^13^

The placement of endografts in the aortic branches has been reported to alter flow patterns in the implanted region,^14^^,^^15^ which, in turn, can have a significant impact on long-term device durability as well as on the prognosis for the patient. Yet, the implications of this TBE geometric configuration on the LSA hemodynamics have not been well-investigated. The integration of medical imaging data and computational modelling allow blood flow simulation under patient-specific conditions.^16^ This study aimed to explore the impact of TBE implantation on the LSA hemodynamics by comparing near-wall hemodynamic parameters before and after the TBE implantation using computational fluid dynamics (CFD) simulations.

## Methods

### Patients and device

Deidentified computed tomography imaging data from 11 patients who had undergone successful implantation of the Gore TBE (W. L. Gore & Associates, Flagstaff, AZ) as part of an industry-sponsored feasibility clinical trial (NCT02777593) were used. For each patient, anonymized Digital Imaging and Communications in Medicine datasets of the preimplantation and postimplantation computed tomography scans (<30 days postoperatively) were analyzed and archived. Inclusion criterion was good quality preimplantation and postimplantation imaging available for the analysis. Because only deidentified data were used, formal institutional review board approval was not required for this limited retrospective study.

The device design has been described in detail previously.^17^^,^^18^ In brief, the Gore TBE is an off-the-shelf, single side-branched modular stent graft system. It comprises a main aortic component and a side branch component. To secure blood flow to the LSA, an 8 mm × 10 mm inner branch or portal is integrated in the main aortic component. Through this portal, the side branch is inserted and sealed. This retrograde design often results in an acute angle of >90° between the portal and the target vessel seal zone of the branch component.

### Three-dimensional geometry reconstruction and mesh generation

**Fig 1:**
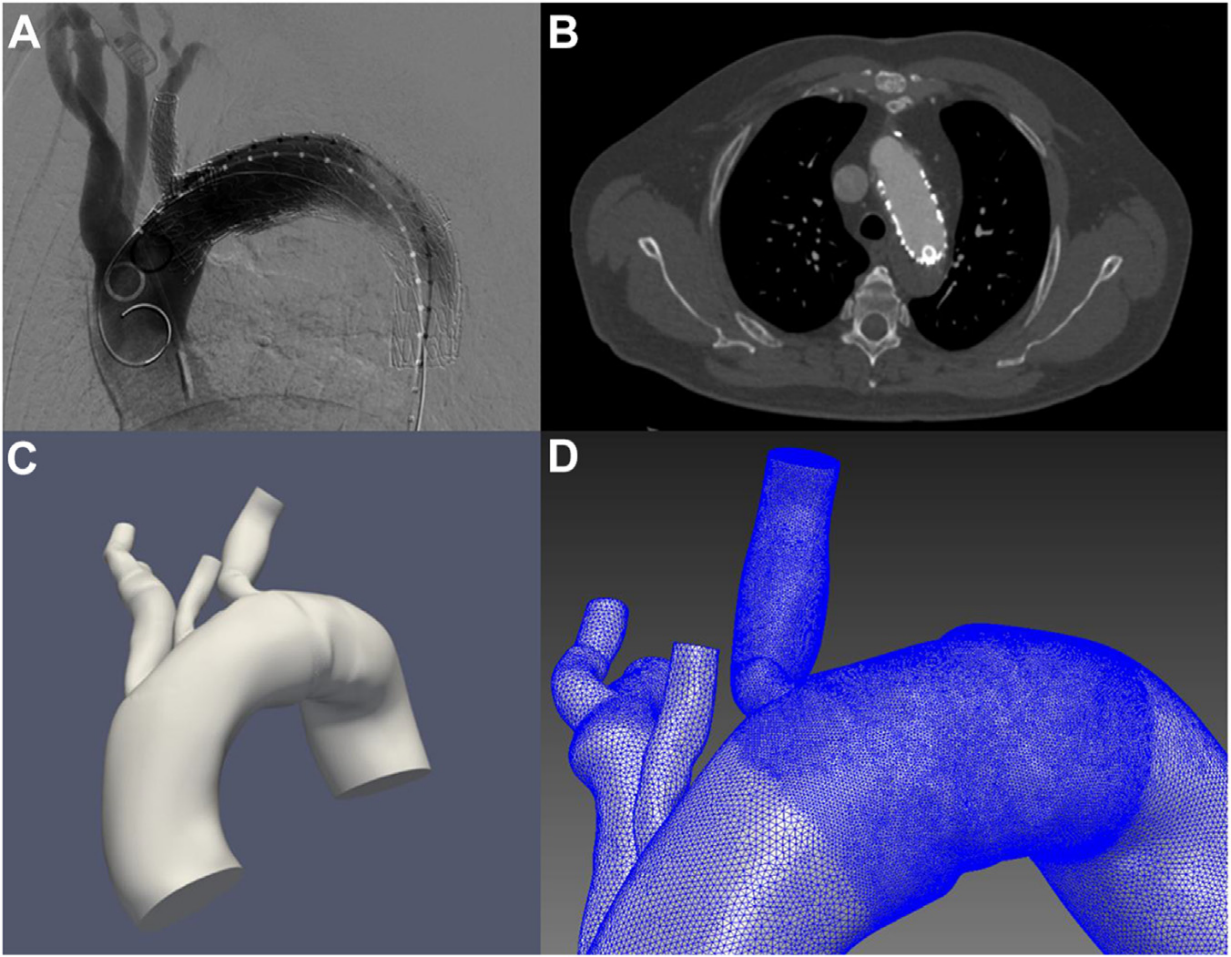
Workflow for three-dimensional (3D) reconstruction and mesh generation. The 3D geometry was reconstructed from computed tomography (CT) angiography. Each reconstructed geometry was discretized into finite element meshes using an open-source mesh generator, TetGen. **(A)** Intraoperative angiography. **(B)** CT Digital Imaging and Communications in Medicine data. **(C)** Reconstructed 3D geometry. **(D)** Mesh.

We acquired 22 deidentified images (11 preimplantation and 11 postimplantation computed tomography images) in a Digital Imaging and Communications in Medicine format that were segmented using the built-in image segmentation toolkit included in an open-source software package SimVascular (Open Source Medical Software Corp, San Diego, CA) (Fig 1, *A* and *B*).^19^ Three-dimensional geometry models including the arch branches were produced from the segmented images (three-dimensional geometry reconstruction).^20^ The final model included segmented aorta above the level of the aortic sinus to the level of the distal aortic arch (Fig 1, *C*). Each reconstructed geometry was then discretized into tetrahedral finite element meshes using an open-source mesh generator, TetGen (Weierstrass Institute for Applied Analysis and Stochastics, Berlin, Germany), which was integrated into SimVascular (Fig 1, *D*).^21^ Each finite element represents a discrete space that presents the flow locally.^22^

### Boundary conditions and flow simulation

Flow simulations were performed on both preimplantation and postimplantation geometric models numerically solving the Navier-Stokes equation, which is the governing equation of fluid behavior, on each finite element of the mesh. Because patient-specific blood flow information was not available, a volumetric flow rate (mean cardiac output of 4.6 L/min) with normal flow waveform was mapped to the inlet using a parabolic profile.^23^^,^^24^ The inlet boundary condition included only the velocity component in the axial direction. As outflow boundary conditions, the three-element Windkessel model with previously published values were applied. The three-element Windkessel model captures the characteristics of the distal vascular bed, proving physiological outflow boundary conditions.^25^ The same boundary conditions were imposed on all simulations for comparison purposes. Blood was modeled as an incompressible, laminar, and Newtonian fluid with a constant density of 1060 kg/m^3^ and viscosity of 0.004 Pa•s.^24^ The wall of the arteries and of the stent graft were modeled as rigid and nondeforming, with a zero-velocity boundary condition at the fluid-wall interface. In other words, at an arterial wall boundary, blood has zero velocity relative to the arterial wall, which corresponds with a no-slip condition.

The mesh independence test for the simulations were carried out to determine the minimum grid resolution to ensure that the results of the analysis are not affected by changing the size of the mesh (also known as a converged mesh). Based on the mesh convergence results,^22^ we set mesh size to 0.5 mm in the areas of interest (aortic arch at the level of the LSA and the LSA) and areas outside the region of interest was meshed to 1 mm. Another point considered was that the resolution of the mesh must be such that it captures the complex flow patterns at the entry of the inner side branch. To do this, we selectively increased mesh refinements at these critical areas allocating more elements with a minimum mesh size of 0.5 mm.^26^ ParaView, which is an open-source visualization software, was used to extract and calculate relevant hemodynamic quantities such as velocity, pressure, wall shear stress (WSS), and the oscillatory shear index (OSI).

### Hemodynamic parameter analysis

To assess the effect of the Gore TBE implantation in the LSA, data were presented on the stented portion of the LSA. For this study, all models were run with the same inflow and outflow boundary conditions. Using this comparative approach, we were able to quantify hemodynamic alterations induced by the TBE design based on the physical laws of fluid dynamics. Hemodynamic parameters used in the analysis were: pressure, flow rate, time-averaged WSS (TAWSS), OSI, and percent area with an OSI of >0.2.

### Measurement definitions

#### WSS

WSS, defined as the tangential drag force of the blood flow acting on the vessel wall, is expressed as force per unit area (dyne/cm^2^). Clinical and experiment evidence suggest that WSS is an important contributor of the development of vascular pathologies by inducing regional variations in endothelial responses.^27^^,^^28^

#### Time averaged WSS

Time averaged WSS (TAWSS) is the time-averaged absolute magnitude of the WSS. TAWSS values of <4 dyne/cm^2^ have been shown to be atherogenic, whereas values of >70 dyne/cm^2^ promotes thrombosis.^29^

#### OSI

The OSI is the WSS oscillations within a cardiac cycle, indicating the WSS vector deflection from predominant blood flow direction. The OSI values can vary between 0 for no deflection (ie, one-directional without oscillations) in WSS vector to 0.5, where the WSS direction changes frequently.^30^ OSI values of >0.25 have been shown to be atherogenic.^31^^,^^32^

#### LSA side branch angulation

The LSA side branch angle was measured between the main aortic component vector and the side branch vector, defined along the main aortic component centerline path and the side branch component centerline, respectively. A straight centerline of the main stent graft measured 0°.

### Statistical analyses

Given the relatively small cohort size, data are presented as median with interquartile range (IQR). Hemodynamic parameters were compared between preimplantation and postimplantation models using the Wilcoxon signed-rank test for two dependent groups (paired data) and with the Mann-Whitney *U* test for two independent groups (unpaired data). A *P* value of <.05 was considered statistically significant for all analyses. All calculations were performed in GraphPad Prism software versions 9.1 for Mac (GraphPad Software, San Diego, CA).

## Results

### Impact of TBE implantation on the LSA pressure and flow rate

Comparing preimplantation and postimplantation results, a small but statistically significant decrease in the mean LSA pressure was observed after implantation: 122.9 (IQR, 122.4-123.8 mm Hg) mm Hg vs 125.1 mm Hg (IQR, 125.0-125.5 mm Hg; *P* = .003). Similarly, there was a statistically significant decrease in the peak LSA pressure: 153 mm Hg (IQR, 151-154 mm Hg) vs 150 mm Hg (IQR, 158-160 mm Hg; *P* = .005). When measuring blood flow, the TBE implantation did not cause a significant change in the mean and peak LSA flow rates: mean LSA flow rate, 13.5 cm^3^/s (IQR, 12.5-14.9 cm^3^/s) vs 12.5 cm^3^/s (IQR, 12.3-13.9 cm^3^/s; *P* = .13); peak LSA flow rate, 41.3 cm^3^/s (IQR, 37.2-44.8 cm^3^/s) vs 40.4 cm^3^/s (IQR, 39.5-41.6 cm^3^/s; *P* = .59). Overall, TBE implantation did not show critically large pressure or flow changes in the LSA, suggesting limited impact on blood flow distal to the LSA.

### WSS-based parameter analysis

**Fig 2:**
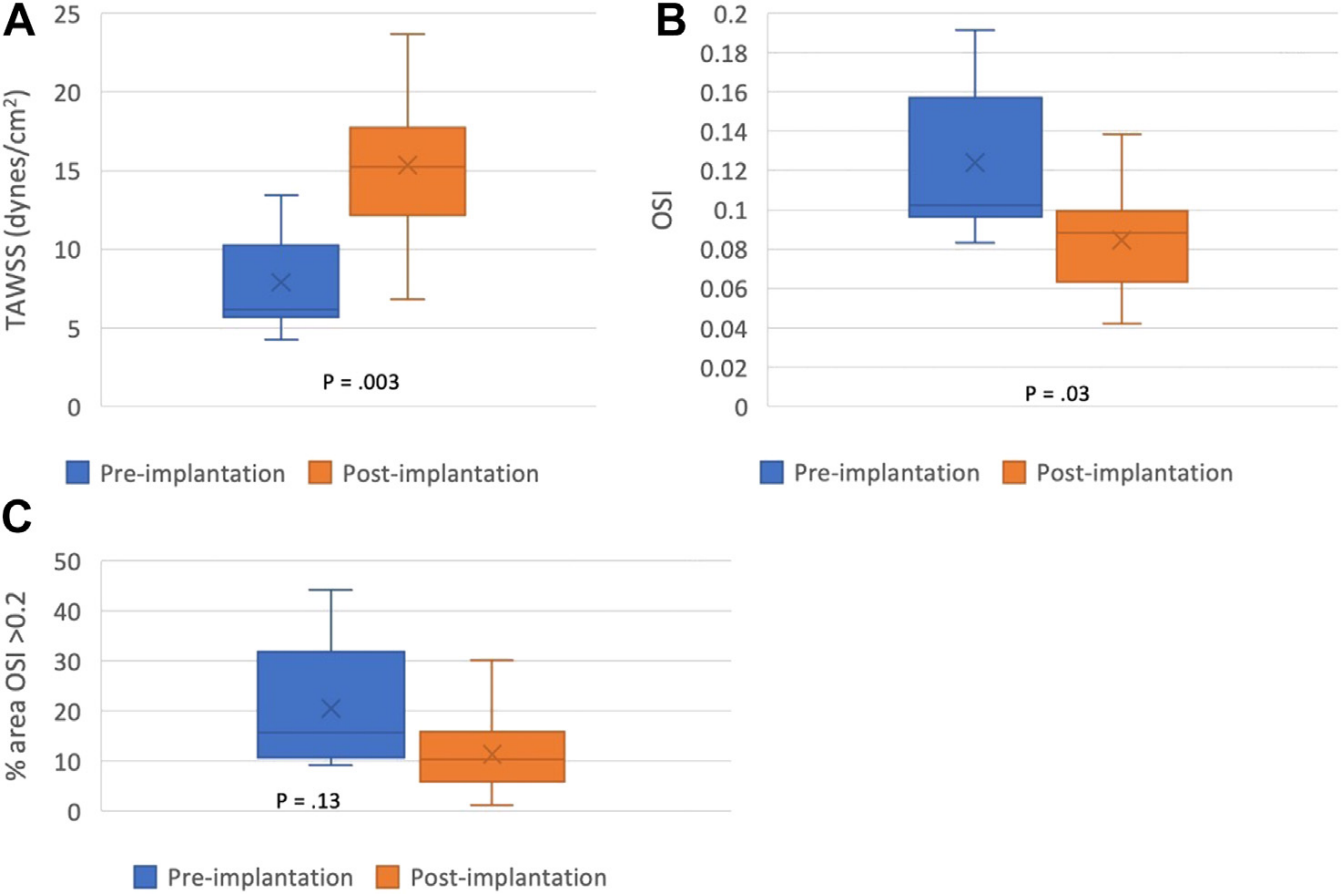
Analysis of near-wall hemodynamic metrics before and after implantation. **(A)** Time-averaged wall shear stress (*TAWSS*). **(B)** Oscillatory shear index (*OSI*). **(C)** Percent area OSI >0.2.

Quantitative analysis showed significant differences in TAWSS and OSI values between before and after implantation. There was a significant postimplantation increase in TAWSS (15.2 dynes/cm^2^ [IQR, 12.2-17.7 dynes/cm^2^] vs 6.2 dynes/cm^2^ [IQR, 5.7-10.3 dynes/cm^2^]; *P* = .003) and a significant decrease in the OSI in postimplantation models (0.088 [IQR, 0.063-0.099] vs 0.1 [IQR, 0.096-0.16]; *P* = .03). The resultant increased TAWSS and decreased OSI led to a trend toward a lower percent area of OSI >0.2: before implantation, 15.7% (IQR, 10.7%-31.9%) vs after implantation, 10.4% (IQR, 5.8%-15.8%; *P* = .13). Fig 2 shows a comparative analysis of near-wall hemodynamic metrics before and after implantation.

**Fig 3:**
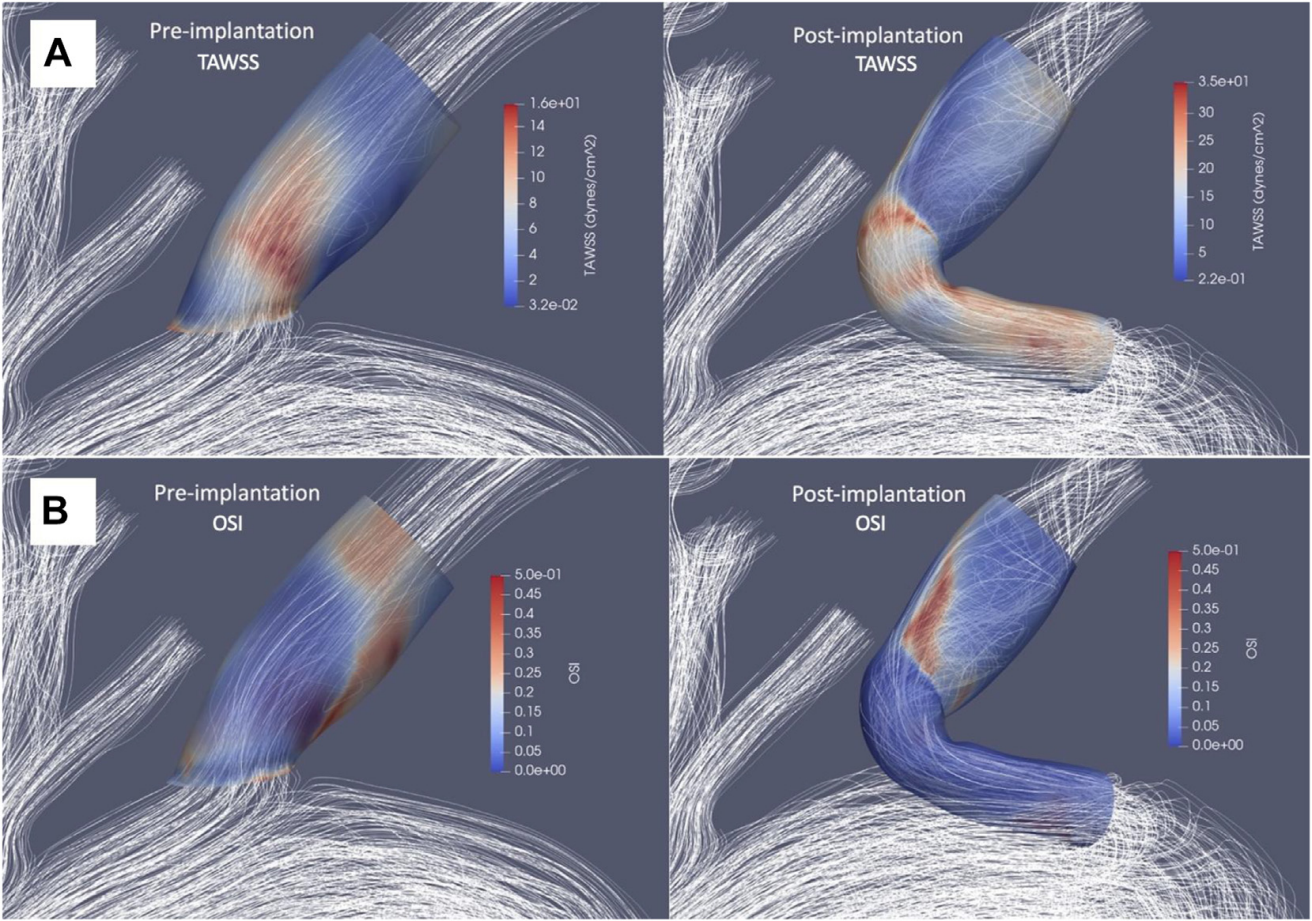
Colorimetric maps of **(A)** time-averaged wall shear stress (*TAWSS*) and **(B)** oscillating shear index (*OSI*) comparing before and after implantation of the thoracic branch endograft (*TBE*) with a view of blood flow streamlines. In the preimplantation images, only the left subclavian artery (LSA) limb is visualized, and the LSA limb plus inner tunnel portion of the arch is visualized in the postimplantation images.

Fig 3 shows preimplantation and postimplantation TAWSS and OSI distributions. The preimplantation characteristic TAWSS regions were located mainly at the ostia of the LSA. After implantation, there seemed to be a moderate amount of TAWSS increase along the entire length of the internal side branch. The spatial distribution of TAWSS and OSI remained qualitatively similar in all cases.

### Influence of the side branch angulation and compression on the hemodynamic performance

**Fig 4:**
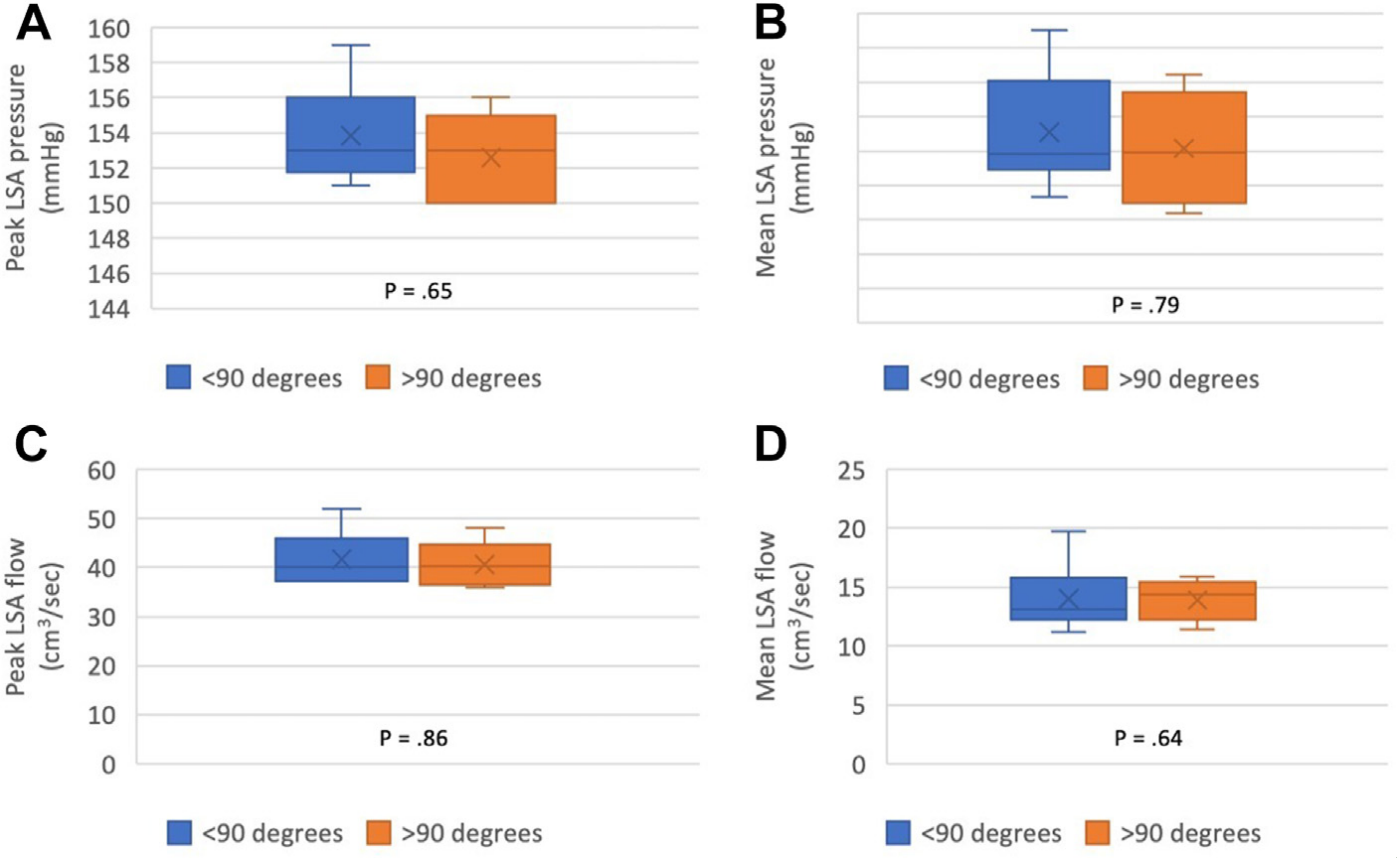
Analysis of the influence of left subclavian artery (*LSA*) side branch angulation (<90° cases vs >90° cases) on **(A)** peak LSA pressure, **(B)** mean LSA pressure, **(C)** peak LSA flow, and **(D)** mean LSA flow.

The influence of LSA side branch angulation as well as compression on the hemodynamic performance was also analyzed and compared in terms of pressure, flow rate, and TAWSS (Table). LSA side branch angulation, defined as the angle between the centerline of the main aortic stent graft and the centerline of the LSA side branch, ranged from 58° to 157° (median, 81°; IQR, 77°-109°). When comparing the angulation cases with <90° vs ≥90°, the differences in the peak and mean pressures (*P* = .65 and *P* = .79, respectively) were not statistically significant (Fig 4, *A* and *B*). There were also no statistically significant differences in the peak and mean flow rates (*P* = .86 and *P* = .64, respectively) (Fig 4, *C* and *D*). Additionally, observed LSA side branch lumen compression (stenosis) ranged from 16% to 57.7% (median, 32.1%; IQR, 20.8%-50%). When simulations of the cases with ≥50% (ie, 50%-58%) compression were compared with those with <50% compression, no significant differences were observed in regard to pressure (peak pressure, *P* = .15; mean pressure, *P* = .13), flow rate (peak flow rate, *P* = .47; mean flow rate, *P* = .94), TAWSS (*P* = .41), and percent area wit an OSI of >0.2 (*P* = .81). As shown in the Table, the findings showed that neither TBE angulation nor compression in the observed ranges seemed to have an impact on the pressure, flow rate, TAWSS, or percent area with an OSI of >0.2.

**Table:** Effect of left subclavian artery (*LSA*) side branch angulation and compression status on hemodynamic parameters

	LSA side branch angulation			LSA side branch compression		
	<90°	≥90°	*P* value	<50%	≥50%^a^	*P* value
Pressure, mm Hg						
Peak	153 (151.8-156)	153 (150-155)	.65	153.5 (152.3-155.8)	151 (150-153)	.15
Mean	122.9 (122.7-124.1)	122.9 (122.2-123.9)	.79	123.3 (122.9-124.1)	122.4 (122.3-122.9)	.13
Flow rate, cm^3^/s						
Peak	39.9 (37.2-45.9)	40.1 (36.6-44.7)	.86	41.9 (37.6-47.2)	41.3 (37.2-41.5)	.47
Mean	13.1 (12.2-15.8)	14.4 (12.2-15.4)	.64	12.9 (11.4-14.8)	13.5 (12.5-14.4)	.94
TAWSS, dynes/cm^2^	15.9 (12.1-18.4)	14.3 (11.9-19.8)	.79	14.1 (11.8-17.5)	15.9 (15.2-20.6)	.41
%A OSI >0.2	12.8 (5.4-16.5)	6.3 (3.5- 21.3)	.65	8.3 (3.8-16.9)	15.2 (6.3-15.8)	.81

*%A OSI >0.2*, Areas with an OSI of >0.2; *TAWSS*, time-averaged wall shear stress.

a Cases with 50%-58% compression. Values are median (interquartile range). *P* values are based on Mann-Whitney-Wilcoxon test.

## Discussion

Implantation of branched endografts in the aortic arch can lead to alterations in flow patterns in the region. TBE implantation in the present study caused a small decrease in the LSA pressure, although there was a trend toward an increased LSA flow rate. The most probable explanation for decreased pressure in the LSA may be the tapered side branch configuration, in that the diameter of the proximal segment within the inner portal is narrower than the diameter of the distal sealing segment. As flow emerges from the inner portal, an expansion in cross-sectional area occurs. This phenomenon, with a change in flow direction, can lead to a diverging passage as flow is directed into the LSA, resulting in a decreased pressure. Sengupta et al^16^ studied the effect of inner portal/branch diameter. Using geometric models of patients who underwent TEVAR with a double inner branch endograft for arch pathology, they found that narrower inner branches tend to cause accelerated flow into the larger respective arch branches, creating a diverging passage as flow is directed into the respective branch artery.

In the present study, the increased blood flow impacted the vessel walls, resulting in increases in TAWSS. Because the OSI has the time-averaged magnitude of WSS in the denominator,^32^ accompanying OSI values were found to be decreased by 12%. TAWSS and OSI are hemodynamic parameters that are used commonly as metrics to evaluate the development and progression of thrombus formation.^33^ Arzani et al^34^ performed patient-specific CFD modeling of 10 abdominal aortic aneurysms to investigate relations between hemodynamics and thrombus progression. Their study showed that a high-shear thrombus forms rapidly and is distinct from the slow formation of coagulation that occurs in stagnant blood.^34^ Another study carried out by Tran et al^22^ explored differences in aortic hemodynamics in 36 patients with juxtarenal abdominal aortic aneurysm treated with fenestrated EVAR who had experienced thrombotic complications during the follow-up period. They found that patients with thrombotic issues had negative postimplantation TAWSS changes. In the present study, all measured postimplantation TAWSS values were within the physiological range of 10 to 70 dyne/cm^2^ (15.2 dyne/cm^2^; IQR, 12.2-17.7 dyne/cm^2^) and with positive postimplantation TAWSS changes (ΔTWASS, +7.4 dyne/cm^2^; IQR, 4.4-9.1 dynes/cm^2^). Additionally, it is generally believed that a high OSI (>0.25) is associated with thrombosis risk.^29^^,^^35^ All postimplantation OSI values in the present study were <0.25 (0.088; IQR, 0.063-0.099). Together these findings suggest that there is no major risk of thrombus formation within the LSA side branch.

Concerns about the risk of thrombosis resulting in occlusions of the side branches after branched TEVAR are valid. Blood clots are formed generally during rapid changes in shear stress.^36^ It is expected that a retrograde configuration, the design characteristic of TBE inner side branch, would impede the flow to the LSA, generating an oscillatory flow that can promote thrombosis. By comparison, an antegrade configuration would generate less resistance to flow. Interestingly, previous reports showed that this is not the case when the length of the side branch is as short as the lengths applicable to branched stent grafts (ie, short enough not to generate resistance to flow). Sutalo et al^37^ evaluated flow characteristics for branch vessel inflow with either an antegrade or a retrograde orientation of the side branch. Using both CFD assessment and benchtop flow rate assessment in an abdominal aortic model with a branch, they showed there are negligible differences in the outflow to a branch vessel in antegrade and retrograde configurations for short conduits. Kandail et al^38^ also compared antegrade and retrograde branch graft designs with fenestrated graft designs and a found negligible difference in the flow rates. In addition, Tricarico et al^39^ demonstrated that, despite antegrade configurations decreasing local LSA resistance, a longer protrusion of the inner branch into the aortic component created flow disturbances with the pulsatile inflow that could induce platelet activation and thrombus formation. To address this particular issue, the TBE inner side branches are anchored to the upper surface of the main aortic component.

The simulation findings of the present study are in congruence with a previous report by Dake et al^12^ on favorable patency and durability with the TBE. In that study, clinical outcomes from an IDE feasibility study of the TBE for zone 2 aortic aneurysms in 84 patients were reported. Through 12 months of follow-up, there were no cases of loss of patency of aortic or LSA side branch component, in addition to no device-related thromboembolic events. However, type I (n = 3) and III (n = 5) endoleaks occurred. Notably, of five type III endoleaks, three involved the connection between the side branch component and the inner portal of the aortic component. According to a study by de Niet et al,^40^ the angle of the branch stents changes over time. During median follow-up of 5 years after branched and fenestrated endovascular aneurysm repair, they found the angulation between directional side branch and bridging stent became 4° more angulated (IQR, −14° to +2°), resulting in 26% (12 of 47) branch occlusion and development of 70% to 99% stenosis in 11% of branches (5 of 47). As such, the type III endoleak observed by Dake et al might be due to the geometric changes over time in bridging stents. All in all, modest hemodynamic disturbances induced by the TBE implantation are unlikely to result in clinically relevant changes.

Given that the retrograde design often results in an acute angle of >90° between the portal and the target vessel seal zone of the branch component, we also explored durability issues with respect to the degree of angulation and compression of the LSA side branch. Within the observed range of 58° to 157°, as shown in the Table, the degree of LSA side branch angulation has insignificant influence on near-wall hemodynamic parameters. Furthermore, the side branch compression from the side branch angulation ranged from 16% to 58% and did not seem to affect the LSA hemodynamics. Taken together, these findings indicate that the TBE is highly adaptable to the variation of the target vessel's location.

van Bakel et al^41^ investigated the hemodynamic impact of TEVAR in proximal landing zone 2 in four patients who underwent carotid-subclavian bypass within the same procedure. The mean flow in the left common carotid artery increased from 0.21 L/min to 0.61 L/min (a 294% increase). This finding suggests blocking the LSA and redirecting blood flow via carotid-subclavian bypass increases flow in the proximal left common carotid artery, which is likely to alter endothelial shear stresses that are known to play important role in the formation of thrombus and development of atherosclerosis.^42^ In contrast, the present study using TBE showed no significant changes to the LSA blood flow. Hence, although the simulations only included the LSA, it is reasonable to assume the blood flow in the supra-aortic branches in the current models remains unaffected. The difference here is that the TBE is designed to adapt to natural anatomy preserving preimplantation aortic morphology as opposed to the extra-anatomical reconstruction with carotid-subclavian bypass. Considered altogether, it seems that the closer a revascularization scheme/endograft design is to preserving preoperative aortic morphology, the smaller the postoperative hemodynamic disturbance will be. In this regard, TBE creates more favorable hemodynamics than carotid-subclavian bypass. However, as yet, long-term results of TBE are unknown.

The present study is limited by its small sample size. Another important limitation is the lack of patient specificity for the boundary conditions, which may affect the accuracy and generalizability of the simulation. However, because the analysis was focused on comparing preimplantation and postimplantation hemodynamic changes, assigning a set of normalized and previously studied conditions extracted from the literature was valid. In addition, the simulations were carried out by assuming the aortic wall to be rigid, although the motion of the aorta itself may also be a significant factor in hemodynamics. The computational model can be improved further by incorporating wall distensibility along with patient-specific inflow and outflow clinical data.

## Conclusions

The implantation of TBE generates modest hemodynamic disturbances that are unlikely to result in clinically relevant changes to the stented LSA environment. The current TBE design seems to be highly tolerant of the local anatomical variations that might influence side branch angulation and compression. Further research with a larger cohort and correlation with clinical outcomes is planned.

## Author Contributions

Conception and design: WY, KM, SH, AW

Analysis and interpretation: WY, KM, CL, JC, AW

Data collection: WY, SH

Writing the article: WY

Critical revision of the article: WY, KM, SH, CL, JC, AW

Final approval of the article: WY, KM, SH, CL, JC, AW

Statistical analysis: WY

Obtained funding: Not applicable

Overall responsibility: WY
